# Ultraviolet light preferences in a white and a brown layer pullet strain

**DOI:** 10.1016/j.psj.2026.106825

**Published:** 2026-03-20

**Authors:** Malou van der Sluis, Jerine A.J. van der Eijk, Thea G.C.M. van Niekerk, Marjaneh Taghavi, Dennis E. te Beest, Maaike Wolthuis-Fillerup, Henk Gunnink, Ingrid C. de Jong

**Affiliations:** a Animal Breeding and Genomics, Wageningen University & Research, 6700 AH Wageningen, the Netherlands; b Animal Health and Welfare, Wageningen University & Research, 6700 AH Wageningen, the Netherlands; c Biometris, Wageningen University & Research, Wageningen, 6700 AA Wageningen, The Netherlands

**Keywords:** Poultry, Welfare, Computer vision, UV light, Behavior

## Abstract

Adequate lighting in laying hen houses is of great importance for hens’ health, welfare and performance. One light aspect that is present in the natural environment of chickens is ultraviolet (**UV**) light, and several studies have suggested that addition of UVA light might have positive effects on laying hens’ welfare. In this study we investigated the preference of layer pullets of two breeds – Lohmann LSL Classic (white; **W**) and Lohmann Brown Classic (**B**) – for two levels of UVA light, and assessed 1) under which light condition (low UVA level (**low-UV**) versus a higher UVA level (**high-UV**) light) they spent most time and 2) what behaviors were performed in the different light conditions. A total of 132 W and 126 B birds were housed in pens (21-22 birds per pen) that were divided in two equal areas with the same light intensity (± 100 lux) but with different UVA levels (low-UV versus high-UV). Video recordings were made from above the pens and birds were automatically counted on each side using a computer vision detection algorithm. Behavior was observed at pen level using instantaneous scan sampling at 1, 2, 4, 6, 8 and 10 weeks of age. For the daytime period, it was observed that W birds had a preference for high-UV in the first three weeks of life, while no clear preferences were observed in B birds. In the last hour before lights off and the first hour of the dark period, B showed a preference for low-UV in week 2 and no clear preference at later ages, while W birds did not show a clear preference at the end of the day at any age. No differences between the low-UV and high-UV light condition were observed in the proportion of behaviors performed in each. These findings suggest that there might not be an exclusive preference for either low-UV or high-UV.

## Introduction

Adequate lighting in laying hen houses comprises light schedule, intensity, spectrum and source. Besides the importance of light for egg production (Bédécarrats and Hanlon, 2017), it is also of great importance for hens’ health and welfare. For example, studies have shown that light aspects may have an effect on body weight (i.e., growth; Muir et al., 2024; Poudel et al., 2022), immune performance (Wei et al., 2022), and bone quality, strength and formation (Sadvakassova et al., 2022; Wei et al., 2022; Clark et al., 2024).

One light aspect that is present in the natural environment of chickens is UV light. Although chickens can see the UVA range (315-400 nm; Prescott and Wathes, 1999b; Rana and Campbell, 2021), lights in commercial poultry houses mostly lack this part of the spectrum (Rana et al., 2024). It has been shown that the addition of UVA light can affect both pullet and adult laying hen behavior, including activity levels, feeding behavior and fearfulness (see review by Rana and Campbell (2021)). For example, Wichman et al. (2021) studied Bovans Robust laying hen pullets in three light environments from 6 to 21 weeks of age: 1) white light without UV (control), 2) a combination of UV and white light (daylight spectrum), and 3) a combination of UV, blue, green and red (forest). They observed more active behaviors (including locomotion, standing and foraging) in the light treatments that included UV than in the control light. Sobotik et al. (2020) studied the effects of adding UVA to LED lighting in White Leghorn hens of 18-72 weeks of age. Compared to the control birds, for whom no UVA was added to LED lighting, they observed faster righting during tonic immobility tests and less intense flapping during inversion tests for the UVA birds at 44 and 72 weeks of age, indicating lower levels of fearfulness. Moreover, they observed lower plasma corticosterone concentrations, heterophil:lymphocyte ratios, and composite asymmetry for the UVA birds, indicating lower stress levels. This highlights that the addition of UVA light might have positive effects on laying hens’ welfare. However, before routinely implementing UVA light in practice, it is important to have insight into what the birds themselves prefer.

Several studies examined pullet and adult laying hens’ own preferences for UV light. For example, in the earlier mentioned study by Wichman et al. (2021) preference tests were performed between 16 and 24 weeks of age, that showed that the birds preferred the forest light (including UV) over the control light (no UV). Rana et al. (2021) studied adult ISA Brown laying hens in a preference test and observed that the hens spent more time in low intensity UVA + UVB light, medium intensity UVA + UVB light and medium intensity UVA light (intensities are total light intensities, simultaneously affecting the UV-index) compared to UV-deficient control light. Liu et al. (2018) provided Hy-Line W-36 chicks with a free-choice preference test, from zero to 8 days old, in which a control lighting (LED with 0% UVA) was tested against LED + UVA at different levels (5%, 10% or 15%). They observed a preference for the 15% UVA compared to the control, but observed no preference in the 10% UVA versus control scenario and observed a preference for the control light when contrasted with 5% UVA.

Given that experiences during rearing can impact laying hens’ later life (see review by Janczak and Riber (2015)), the light preferences of pullets are important to take into account. It is, however, yet unclear what layer pullets’ preferences are regarding UV light across different times of the day and whether these preferences differ between genetic lines. Therefore, in this study we investigated the preference of layer pullets of two breeds – a white (Lohmann Selected Leghorn Classic (**W**)) and a brown breed (Lohmann Brown Classic (**B**)) – for two levels of UVA light, and assessed 1) under which light condition (low UVA level versus a higher UVA level) they spent most time and 2) what behaviors were performed in the different light conditions. We hypothesized that the pullets would prefer the higher UV level light condition during the day and the low UV level light condition at the end of the day, as this might resemble the natural environment of chickens. Moreover, we hypothesized that more active behaviors would be shown in the higher UV light condition, based on the earlier-discussed observations for older birds (Wichman et al., 2021). The results of this study can help to improve lighting programs for layer pullets, to match the birds’ preferences and hereby potentially improve their welfare in production systems.

## Materials and methods

### Ethical statement

This study was carried out at the experimental facility of Wageningen Bioveterinary Research (Lelystad, the Netherlands). The housing, management and experimental procedures were conducted in accordance with the national legislation on animal welfare and animal experiments, and were approved by the institutional Animal Welfare Body. Because the procedures were non-invasive, this study was not considered to be an animal experiment under the Law on Animal Experiments, as confirmed by the institutional Animal Welfare Body (12th of July, 2022, Lelystad, The Netherlands).

### Housing and birds

Day-old layer chicks of two breeds, Lohmann Selected Leghorn Classic (W) and Lohmann Brown Classic (B), were obtained from a commercial hatchery (Ter Heerdt, Zevenaar, the Netherlands), where they were incubated in the same way in complete darkness and without feed access according to commercial procedures. A total of 132 W and 126 B hens were randomly allocated across pens within two rooms, with 22 W or 21 B hens per pen (stocking densities of 5.7 and 5.5 birds/m^2^, respectively) and a total of 6 pens per breed. These floor pens had a size of 3.84 m^2^ (length 3.2 m, width 1.2 m and height 1 m), and were divided in 2 equal areas (each 1.92 m^2^; length 1.6 m and width 1.2 m) with the same light intensity in each area (± 100 lux) but with different levels of UVA light (see section **Light**). Both areas had wood shavings as litter and included a pan feeder and nipple drinkers with cups. Furthermore, a bucket with lucerne and a perch were provided in each area. Pens included a net on top from 4 weeks of age onwards to avoid hens from escaping to other pens. The birds had *ad libitum* access to feed and water. A two-phase feeding schedule was applied, with rearing diet 1 from day 0 to 35 (crumble) and rearing diet 2 from day 35 to 70 (meal). Both diets were produced by ABZ Diervoeding (Leusden, the Netherlands). Chicks were vaccinated against Newcastle Disease, Infectious Bursal Disease, Marek’s disease and Infectious Bronchitis at the hatchery. Chicks were (again) vaccinated against Newcastle Disease at 4 weeks of age via spray, against Infectious Bronchitis at 8 weeks of age via eye drops and against *Salmonella Enteritis* at 6 weeks of age via intramuscular injection. The rooms in which the pens were located had no windows and were climate controlled with 34°C at arrival, after which the temperature gradually decreased to a constant temperature of 19°C from 8 weeks of age onwards. The birds were monitored in the pens up to 70 days of age. All birds were marked with a purple dot using animal identification dye on the head/neck area for easier recognition on video recordings. The birds were furthermore weighed per group at placement, at 4 weeks of age and at the end of the experiment, and mortality was noted daily at pen level.

### Light

Within each pen the same light intensity was provided (± 100 lux) but one half of the pen received a higher level of UVA light (i.e., the higher UV light condition; **high-UV**) whereas the other half of the pen received a lower level of UVA light (i.e., the low UV light condition; **low-UV**). The location of the light condition within the pen was randomly allocated per day throughout the experiment, to avoid that a preference for level of UVA light was entwined with location. Areas were separated from each other using a black ‘lightproof’ plastic sheet, which was also used on the sides of the pens, but the tops remained open. In the center there was an opening (width 30 cm and height 30 cm) to allow the birds to freely move from one half of the pen to the other half. The lights were installed approximately 1 m above the pen floor, and consisted of the Optilima V2 All light for the high-UV condition, with a specified UV level of 7-8%, and the Optilima Only Sunlike light for the low-UV condition, with a specified UV level of close to 0%, both from Big Dutchman. The light intensity at chick height (± 30 cm, pointing the sensor upwards) was measured using MAVOspec BASE in half of the pens. On average, the light intensity in the pens was 96.9 lux for the low-UV condition and 98.4 lux for the high-UV condition (i.e., similar overall light intensities were maintained and only the level of UV was varied between the two conditions). The measured intensities in the center and corners of the pen, as well as the light spectra for the low-UV and high-UV condition are presented in Supplementary data 1. The lighting schedule was 23L:1D from day 0 to 2, 16L:8D from day 3 to 7, 15L:9D in week 2, 14L:10D in week 3, 12L:12D in week 4, 11L:13D in week 5, 10L:14D in week 6 and 9L:15D from week 7 onwards. A twilight period of 10 minutes when transitioning from light to dark and vice versa was used. Lights were on from 08.00 h in the morning and went off after the above-described light period duration for the different ages of the birds.

Unfortunately, we experienced some issues with the lights due to power shortages, and therefore data were excluded from 18 to 24 d of age for room 1 (pens 1-6) and for 23 d of age for room 2 (pens 7-12). In room 1, the lights did not turn off on one side of the pen for 18 to 21 d of age. The lights that remained on were different from the low-UV and high-UV condition, as it was more yellow-green light. The lighting on the other side of the pens did continue to alternate between the low-UV and high-UV condition. Moreover, at 22 d of age, both areas had continuous light until at least 23.00 h and lights were on the following day from at least 7.00 h (recordings were only scheduled between 7.00 and 23.00 h, and therefore the exact times are not known). On day 23, a power shortage occurred around 15.00 h. The pens in room 1 had yellow/green light from 15.00 until 21.00 h on one side, which changed back to the normal light aspects and schedule from 21.00 h onwards, whereas the other side of the pens had continuous light until at least 23.00 h and lights were on the following day (i.e., at 24 d of age) from at least 7.00 h. After 10.00 h at 24 d of age the light aspects and schedule were running correctly for this side as well. Room 2 experienced the same power shortage on day 23, and had yellow/green light from 15.00 h until 21.00 h, after which the light aspects and schedule were running correctly again.

### Light preference recordings

To assess the preference of the birds for low-UV versus high-UV light conditions based on where they were present most often, video recordings of the pens were made from above and birds were automatically counted on both sides of the pen using a computer vision (**CV**) detection algorithm. Videos were analyzed for two days per week of age, for the period from one to ten weeks of age, except in week 4 (due to the earlier-mentioned lights issue) and in week 9, in which only one day of data was available. The CV algorithm was implemented from one hour before lights on (so starting at 07.00) until one hour after lights off (which changed as the birds aged). For the first week, the hour after dark was not available, due to that hour being on the next day (i.e., from 00.00 to 01.00). The algorithm counted the birds every five minutes.

**Fig. 1:**
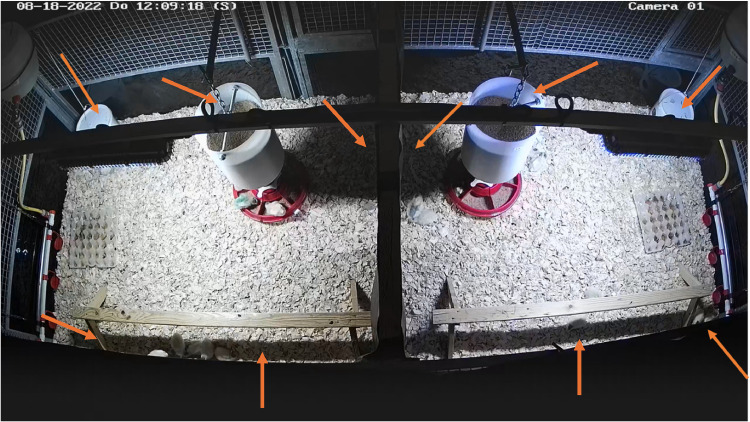
**Example frame from the top-view videos of the pens.** Arrows indicate spots in which the CV detection was complicated or not possible, due to obstacles in sight (bar with light units, feeder) or dark shades produced by equipment in the pen (feeders, perches).

The CV approach was similar to – and is described in more detail in – earlier work (van der Eijk et al., 2025; van der Sluis et al., 2025b). In short, a deep learning object detection model (YOLOv8) was used to detect the birds in the pen. The earlier models from van der Eijk et al. (2025) and van der Sluis et al. (2025b) were used as pre-annotation here, and manual corrections were made where necessary. In total, 300 images for training and 132 images for validation were selected from the current dataset, from weeks 1, 3 and 5, and 212 images were selected for the test set, from all weeks. Table 1 shows the confusion matrix of the model’s performance on the test set. Unfortunately, we experienced some difficulties with objects blocking the view and with shade in the pens (Fig. 1), making it hard to detect birds in those areas. As a result, the detection performance of the model on the full dataset was suboptimal: on average, 34% of the W birds and 43% of the B birds were not detected using the algorithm. Consequently, the results from the CV detection need to be interpreted with some caution. It is, however, important to note that this shade was present in both compartments of the pen and that the position of the UV treatments was alternated daily during the trials, and therefore no systematic error in the detections for a single light treatment is expected.

**Table 1:** Confusion matrix of the model’s performance on the test set.

	True: Chicken	True: Background
Predicted: Chicken	3251	213
Predicted: Background	128	0

### Behavioral observations

Behavior was observed live at pen level using instantaneous scan sampling at six ages, i.e. at 1, 2, 4, 6, 8 and 10 weeks of age. The behavioral observations were performed on one day per age. Within the days of observation, each area (i.e., each side of each pen) was observed twice, once in the morning (09.00 – 12.30 h) and once in the afternoon (13.00 – 16.30 h, to avoid overlap with the onset of the dark period at later ages), in randomized order across pens. Each observation consisted of scoring all areas, where each area was scanned 5 times after a 2.5 min habituation period to allow the birds to habituate to the presence of the observer. Per scan, the behavior of all birds in the area was scored according to the ethogram in Table 2. Behavioral observations were performed by two trained observers, that both assessed half of the pens, with an equal distribution of W and B birds. Subsequently, the five sets of scans per observation were merged into a single record, resulting in two sets of behavioral counts per half of the pen per observation day, except for pens 1-6 for which the measurement at 4 weeks of age was excluded as this was performed on day 24, on which the light situation was incorrect.

**Table 2:** Ethogram used for behavioral observations.

Categories	Behavior	Description
Ingestion	Eating	Having the head above or in the feeder or pecking at feed in the feeder
	Drinking	Pecking at the drinking nipples or cup beneath the drinking nipple
Active	Locomotion	Walking, running, jumping or hopping without performing any other type of behavior
Standing	Standing	Standing without performing any other type of behavior
Inactive	Inactive	Sitting or lying while not engaged in any other activities
	Perching	Perching, including resting, standing, preening or stretching on the perch
Foraging	Explorative pecking	Pecking at the enrichment (hay/lucerne)
	Foraging	Pecking and/or scratching at the ground, litter
Comfort	Preening	Preening (manipulating own feathers with the beak or paws), stretching, wing flaps, feather ruffles, shakes (outside context of dustbathing)
	Dustbathing	Rubs head and body against the ground, pecks and scratches while lying on the side, distributes substrate over body or shakes off substrates from feathers
Social	Feather pecking	Pecking at feathers of conspecifics
	Aggressive pecking	Pecking at the head or comb area of conspecifics
Other	Other	All other behaviors not described above
	Object pecking	Pecking at wall, perch, etc. but not ground, litter, feeder or drinker

### Statistical analyses

All statistics were performed in R version 4.4.1 (R Core Team, 2024) and analyses were conducted using pen as the replicate unit. To examine the distribution of the birds across the low-UV and high-UV side of the pen over time, beta binomial models were fitted using the glmmTMB package (Brooks et al., 2017), with week and/or breed as fixed effects and pen as a random effect. Using the respective relevant subset of data, we examined (1) breed-week interaction effects, (2) the effect of week per breed, and (3) the difference between breeds per week using the model including the breed-week interaction effect. Models were compared using the anova command, and pair-wise contrasts were determined using the emmeans package (Lenth, 2024), using a Tukey p-value adjustment. All analyses were initially performed for the full light period plus the first hour of the dark period for that day (which differed per age, as described earlier). Subsequently, the same analyses were performed for only the early morning (the first hour of the light period) or only the end of the day (the first hour before the dark period plus the first hour of the dark period; this was done to assess where birds were when the lights turned off). In Supplementary data 2, the distributions of birds over time (per hour) and the pen-level variation can be found.

To assess whether there were differences between breeds, ages and UV treatments (low-UV versus high-UV) in what behaviors were mainly performed, beta binomial models were implemented using the glmmTMB package (Brooks et al., 2017) to analyze the counts of each of the behavioral categories from the scan sampling separately. Each model started with breed, age and light treatment and their two- and three-way interactions as fixed effects, and pen as random effect to account for repeated measurements, and subsequently interaction effects that were not statistically significant (*p* > 0.05) were excluded from the model (first for the three-way interaction and then testing for each of the two-way interactions in the presence of the other two-way interactions). In the results, interaction effects are reported when present, and main effects are reported when no significant interaction effects were observed. Pair-wise contrasts for the resulting models were determined using the emmeans package (Lenth, 2024), using a Tukey p-value adjustment. Predicted emmeans-values were also used for plotting the results.

## Results

### Light preferences

#### Light preferences across the day

**Fig. 2:**
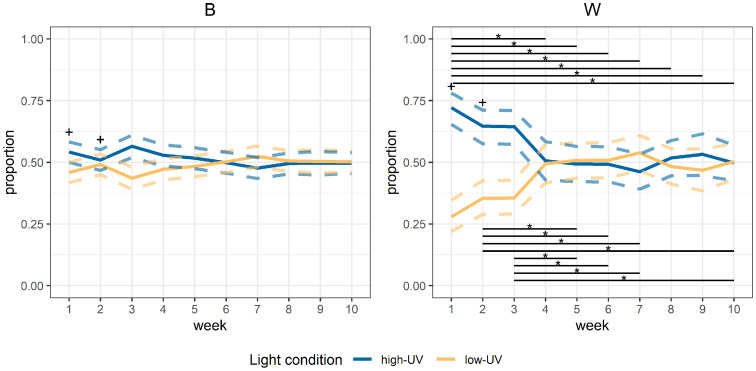
**Estimated proportions of birds present in the different light treatment compartments over time, across the full day (from the start of the light period until one hour after the light period).** Different panels show the different breeds, and the week of age is shown on the x-axis. The solid lines represent the model fit within a breed and the dashed lines indicate the 95% confidence intervals. High-UV = high level of UV light present; low-UV = low level of UV light present; *B* = Lohmann Brown Classic; *W* = Lohmann Selected Leghorn Classic; * = significant differences between weeks within the breed (the start and end of the associated black lines indicate the respective weeks that are compared); + = significant differences between breeds within a week.

For the full day (the light period plus one hour after, except for week 1 when the hour after the light period was not available), the models revealed an interaction between week and breed (*p* < 0.001). Within B birds, there were no differences between weeks (*p* = 0.22; Fig. 2). For W birds, there were differences between weeks (*p* < 0.001), with a clearer preference for high-UV in weeks 1, 2 and 3 than in later weeks (Fig. 2). When comparing breeds within weeks, a difference was observed for weeks 1 and 2 (*p* < 0.001 and *p* = 0.001; Fig. 2), with a stronger preference for high-UV in W birds than in B birds. No differences were observed for weeks 3 to 10.

#### Light preferences in the early morning

**Fig. 3:**
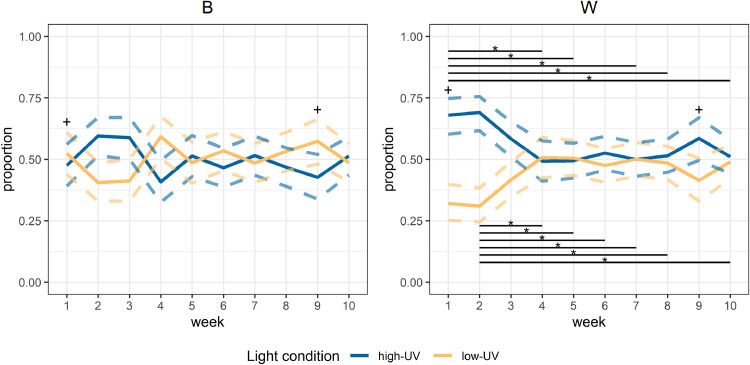
**Estimated proportions of birds present in the different light treatment compartments in the first hour of the light period, over weeks.** Different panels show the different breeds, and the week of age is shown on the x-axis. The solid lines represent the model fit within a breed and the dashed lines indicate the 95% confidence intervals. High-UV = high level of UV light present; low-UV = low level of UV light present; *B* = Lohmann Brown Classic; *W* = Lohmann Selected Leghorn Classic; * = significant differences between weeks within the breed (the start and end of the associated black lines indicate the respective weeks that are compared); + = significant differences between breeds within a week.

When assessing only the early period (the first hour of the light period), the models indicated no statistically significant interaction effect between week and breed (*p* = 0.08). Within B birds, there were no differences between weeks (*p* = 0.07; Fig. 3). For W birds, there were differences between weeks (*p* = 0.001 for the week effect), with a stronger preference for high-UV in weeks 1 and 2 than in later weeks (Fig. 3). When comparing weeks between breeds, a difference between B and W birds was observed in week 1 (*p* < 0.001) and week 9 (*p* = 0.02), with W birds showing a stronger preference for high-UV (Fig. 3). For the other weeks, no difference was observed between the breeds. The overall pattern for the first hour of the light period resembles the pattern observed for full days (Fig. 2).

#### Light preferences at the end of the day

**Fig. 4:**
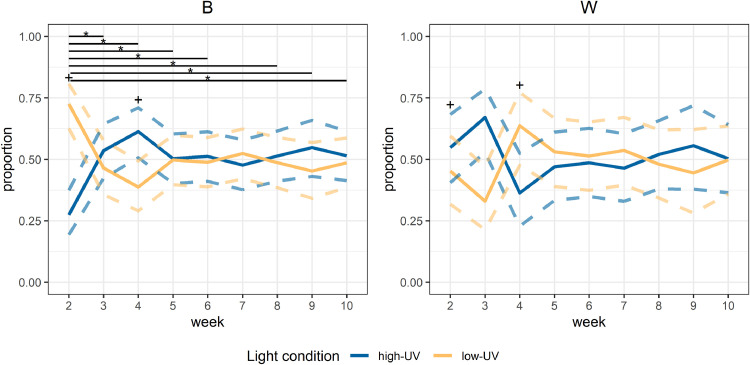
**Estimated proportions of birds present in the different light treatment compartments at the end of the day (one hour before the dark period and the first hour of the dark period), over weeks.** Different panels show the different breeds, and the week of age is shown on the x-axis. The solid lines represent the model fit within a breed and the dashed lines indicate the 95% confidence intervals. High-UV = high level of UV light present; low-UV = low level of UV light present; *B* = Lohmann Brown Classic; *W* = Lohmann Selected Leghorn Classic; * = significant differences between weeks within the breed (the start and end of the associated black lines indicate the respective weeks that are compared); + = significant differences between breeds within a week.

When assessing only the late period (the last hour before the dark period and the first hour of the dark period), the models indicated an interaction effect between week and breed (*p* = 0.03). Within B birds, an overall week effect was observed (*p* = 0.01; Fig. 4), with a stronger preference for low-UV in week 2 than in most later weeks. Within W birds, no week effect was observed (*p* = 0.34; Fig. 4). When comparing weeks between breeds, a difference between B and W birds was observed in weeks 2 and 4 (*p* = 0.003 and *p* = 0.01, respectively; Fig. 4). In week 2, B birds showed a stronger preference for low-UV than W birds, while in week 4 B birds showed a stronger preference for high-UV than W birds. Week 1 was not taken into account, as no data were available on the hour after the light period.

### Pen-level variation in light preferences across the day

**Fig. 5:**
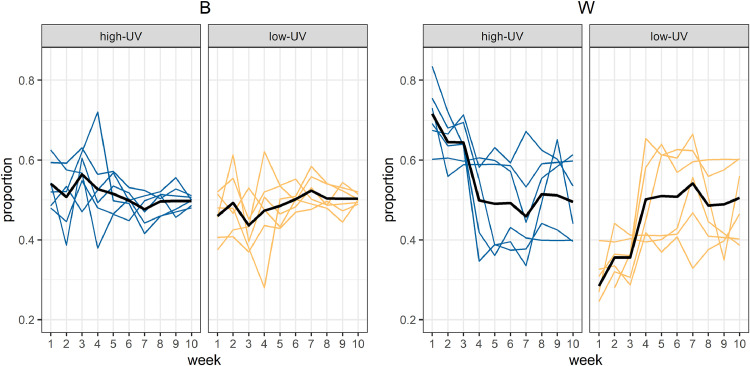
**Proportional bird counts in the two light conditions over weeks, for Lohmann Classic Brown (left) and Lohmann LSL Classic (right), respectively.** Separate panels show the different light treatments, with colored lines representing individual pens. Black lines indicate the mean across pens. High-UV = high level of UV light present; low-UV = low level of UV light present; *B* = Lohmann Brown Classic; *W* = Lohmann Selected Leghorn Classic.

Variation was observed between pens in the proportion of birds in the different light conditions across weeks. Fig. 5 shows the proportional bird counts for both breeds across weeks, and it appears that within W birds the pens start to differentiate from week 4 onwards. Several pens show a stronger preference for high-UV while other pens show a preference for low-UV, which together result in no clear overall preference across pens from 4 weeks of age onwards.

### Behavior in the different light conditions

**Fig. 6:**
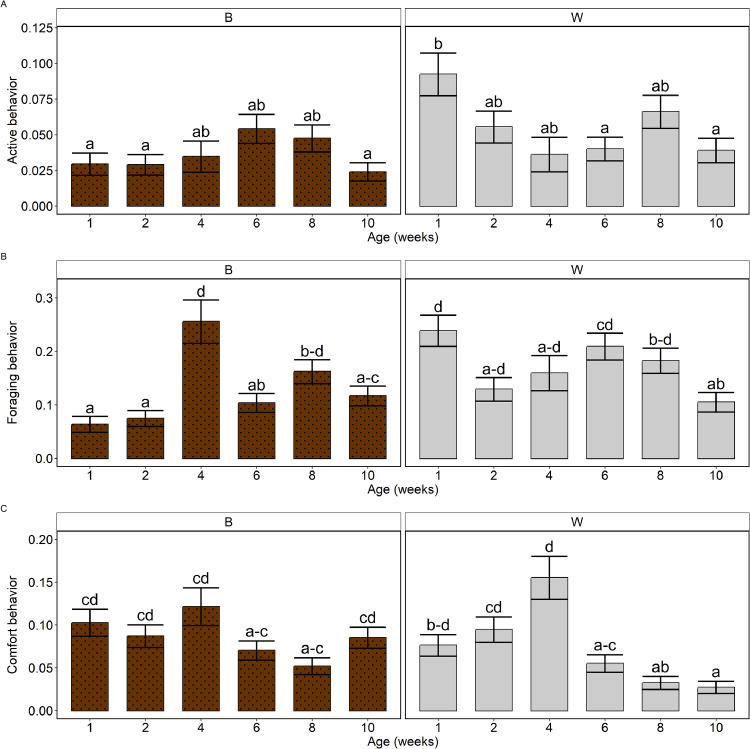
**Estimated proportions of behaviors shown, by breed (panels) and age (x-axis).** A) Active behavior, B) Foraging behavior, C) Comfort behavior. Estimated proportions are based on emmeans-estimations for the breed by age interaction in the fitted models. Error bars indicate standard errors. *B* = Lohmann Brown Classic; *W* = Lohmann Selected Leghorn Classic. ^a–d^ values lacking a common superscript within a behavioral category differ significantly (*p* < 0.05).

The model analyses revealed no differences between the high-UV and the low-UV light condition in the proportions of behaviors shown. When focusing on effects of breed and age, interactions between breed and age were observed for active behavior (*p* = 0.007; Fig. 6A), foraging behavior (*p* < 0.001; Fig. 6B) and comfort behavior (*p* = 0.007; Fig. 6C), when averaged over light treatment. The proportion of active behavior was higher in W birds in week 1 than in B birds in weeks 1, 2 and 10, as well as higher than in W birds in weeks 6 and 10. Regarding foraging behavior, the proportion of foraging behavior was higher in weeks 1 and 6 than in week 10 within W birds. Within B birds, the proportion of foraging behavior was higher in week 4 than in weeks 1, 2, 6 and 10, and higher in week 8 than in weeks 1 and 2. Moreover, the breeds differed from each other in the proportion of foraging behavior in weeks 1 and 6, with more foraging behavior in W birds in both weeks. For comfort behavior, it was observed that W birds in week 4 showed a higher proportion of comfort behavior than W birds in weeks 6, 8 and 10 and than B birds in weeks 6 and 8. Moreover, the proportion of comfort behavior in W birds in week 10 was lower than for W birds in weeks 1 and 2 (and 4), and than for B birds in weeks 1, 2, 4 and 10.

**Fig. 7:**
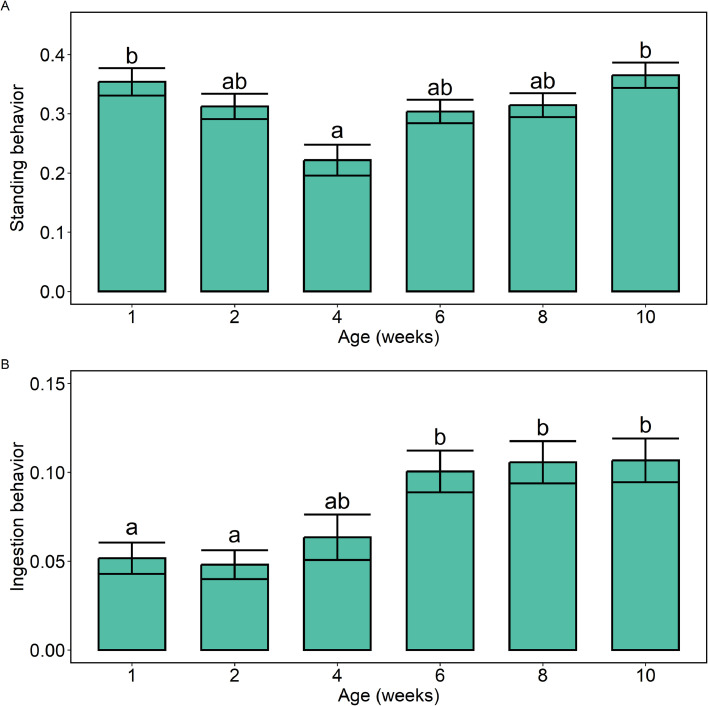
**Estimated proportions of behaviors by age.** A) Standing behavior, B) Ingestion behavior. Estimated proportions are based on emmeans-estimations for age in the fitted model. Error bars indicate standard errors. ^a–b^ values lacking a common superscript within a behavioral category differ significantly (*p* < 0.05).

Furthermore, several main effects were observed. For standing behavior, main effects for breed (*p* < 0.001) and age (*p* = 0.002) were observed, with more standing behavior in B birds than in W birds (averaged over age and light treatment) and more standing behavior in weeks 1 and 10 than in week 4 (averaged over breed and light treatment; Fig. 7A). Furthermore, an effect of age was observed on the proportion of ingestion behavior (*p* < 0.001; Fig. 7B), when averaging across breeds and light treatments, with a smaller proportion of ingestion behavior in weeks 1 and 2 than in weeks 6, 8 and 10. Moreover, there was a breed effect in the proportion of other behavior (*p* = 0.02), with more other behavior in W birds. For inactive behavior and social behavior, no effects of age, breed or light treatment were observed.

## Discussion

In this study, we investigated the preference of layer pullets of two breeds – Lohmann Selected Leghorn Classic and Lohmann Brown Classic – for UVA light, and assessed 1) under which light condition (low-UV versus high-UV light) they spent most time and 2) what behaviors were performed in the different light conditions. Overall, across the light period it was observed that W birds had a preference for high-UV light in the first three weeks of life, while no clear preferences were observed for B birds. No differences were observed in the type of behaviors performed in the high-UV versus the low-UV light condition.

### Light preferences

#### Light preferences across the day

Across the day (from the start of the light period until one hour after the light period), W birds showed a preference for high-UV light in the first three weeks of life, while there was no clear preference later in life. A preference for (more) UV light is in line with observations by Rana et al. (2021). However, Liu et al. (2018) observed a preference for 15% UVA compared to control light without UV, but observed no preference in the 10% UVA versus control scenario and observed a preference for the control light when contrasted with 5% UVA. This contrasts with the observed preference for 7-8% UV in this study. In general, the observed preference for high-UV may be linked to those light conditions being more similar to the natural environment of chickens. Interestingly, in the current study the preference of W birds for high-UV light was only clearly visible in the first weeks of life. Similarly, van der Sluis et al. (2025a) observed a clearer early-life preference for UV light than later in life for broiler chickens. There, it was hypothesized that the birds might have been better able to assess their environment in the presence of UV light, as feathers and several food resources reflect UVA light (Bennett and Cuthill, 1994; Prescott and Wathes, 1999a, 1999b), and that – after assessment of the environment in the first weeks of life – the birds habituated to their environment and UVA light was no longer of great importance. Similar effects might have played a role in the early preference for high-UV observed in W birds in this study, but more research is required to determine this, especially given that other studies (e.g. Rana et al., 2021 (for brown layers)) have observed preferences for UV light also in adult hens. In general, however, several light preference studies (also on light intensity and spectrum) have observed stronger preferences in the first weeks of life in chickens (van der Sluis et al., 2025b; van der Eijk et al., 2025), suggesting that light conditions are potentially more important for chickens when they are young.

In contrast, B birds did not show a clear preference for high-UV at any of the studied ages. Brown and white layer pullets have been shown to differ in some aspects of behavior, such as fear responses (Manet et al., 2023; Rentsch et al., 2024) and perching (Rentsch et al., 2023). Moreover, from a genetics viewpoint, brown and white layers have been shown to have quite distinct breed origins: a study on the genetic diversity and population structure of different chicken populations has indicated that white egg layers clustered with north-west European chickens, while brown layers clustered in an African/south-east European gene pool (Lyimo et al., 2014). Possibly, these differences in behavior and origin also affect UV-light preferences. Brown layers originate from regions with higher UV levels (i.e., UV levels are higher for regions closer to the equator (World Health Organization, 2016)), and these higher UV levels may increase the risk of damage to the skin. It can be hypothesized that brown layers may therefore show less of a preference for UV light than white layers that originate from regions with lower UV levels, that are less likely to damage the skin. Rana et al. (2021) studied light preferences of ISA Brown laying hens for different intensities of UV light (overall light intensities, which also affected the resulting UV index) and observed that for the condition with both UVA and UVB light, the hens preferred the UV light at low and medium intensities, but not at high intensity. They suggest that this might be related to higher UVB levels potentially leading to skin damage. In line with this hypothesis, Stadig et al. (2017) showed a decrease in ranging in free-range slow-growing broiler chickens when the solar radiation was higher. Therefore, careful consideration of – and further research on – the level of UVA light to be implemented is crucial, to avoid potential skin damage in birds or farm personnel. Overall, the differences in origin of brown versus white laying hens may explain the observed differences in the preference for UV light conditions, but this requires further investigation. In future research, also potential differences in UV reflection of white versus dark feathers (see e.g. Bright, 2007) would be interesting to examine further, to assess whether this may impact UV preferences in chickens.

#### Light preferences in the early morning

The light preferences in the early morning resembled the light preferences observed across the day: while there was no clear preference in B birds, W birds preferred high-UV light in the first weeks of life. The main difference (compared to the full day results) that stands out is that in the first hour of the light period in week 9 W birds spent more time in the high-UV light condition whereas B birds spent more time in the low-UV light condition. However, this preference appeared to be numerically small and less strong than in the first two weeks.

#### Light preferences at the end of the day

In the last hour before lights off and the first hour of the dark period, B birds showed a preference for low-UV in week 2 and no clear preference at later ages. In a study on broilers (van der Sluis et al., 2025a), a similar observation was made for the end of the day, in slow-growing broilers of 6 to 8 weeks of age. There, it was hypothesized that this preference for no UV light at the end of the day may be linked to the UV conditions in the natural environment of chickens, with less UV light when night falls (van der Sluis et al., 2025a). However, this hypothesis requires further investigation, especially given that this preference for low-UV was only observed for B birds at two weeks of age, and not at other ages or in W birds. Possibly, the stronger overall preference across the day for high-UV in the first weeks in W birds (which B birds did not show) may compete with a potential preference for low-UV before the onset of the dark period, resulting in no clear preferences at the end of the day, but this remains to be investigated.

#### Pen-level variation in light preferences across the day

The earlier discussed light preference patterns were based on group means across multiple pens. However, individual pens were observed to differ in their distribution across the two light conditions. Within W birds, the pens started to differentiate from week 4 onwards, in which several pens showed a stronger preference for high-UV while other pens showed a preference for low-UV. Given that the light conditions were alternated between days within pens, this appears to not be caused by a side preference. Possibly, individual differences, dominance and/or group interactions play a role in these observed patterns, although it is interesting to note that such a differentiation was not clearly visible for brown pullets. For future research, it would be interesting to mark birds to allow individual identification and assess the group dynamics in the choice for one of the two light conditions.

#### Limitations

It is important to note that the detection performance of the model to assess light preferences was not optimal, as on average 34% of the W birds and 43% of the B birds were not detected using the algorithm across all weeks, which might have impacted the results. However, given that the obstructions and shade that caused the difficulty in detection was present on both sides of the pen and that the light treatments were alternated between the two sides of the pen during the trial, we do not expect this to have resulted in a systematic error in the preference recordings. Furthermore, as reported earlier, issues were experienced with the lights due to power shortages. Even though the time points at which this occurred were excluded from the data this may still have resulted in stress in the birds, the effect of which may have carried over onto the observation days (both for the count data and the behavioural observations). With the UV light condition alternating between the two compartments within the pen, it is not expected that this had more effect for one light condition than for the other, and therefore the comparison between the light conditions can still be informative. It can, however, not be ruled out that the overall observed behavioural frequencies (discussed further on) were affected by the potential stress from the light issues.

### Behavior in the different light conditions

No differences between the high-UV and the low-UV light condition were observed in the proportions of behaviors shown. This was unexpected, given that some other studies have shown differences in behavior between light conditions, although younger birds were included in the current study. For example, Wichman et al. (2021) studied behavior of laying hens in three different light conditions with similar light intensities: white light without UV (mean overall light intensity of 7.9 ± 1.0 lux), light resembling daylight including UV (with a peak at 395 nm; mean overall light intensity of 10.9 ± 1.4 lux), and light resembling the forest (including UV light; mean overall light intensity of 8.3 ± 0.05 lux). They observed – using instantaneous observations in choice experiments in which birds were provided with a choice between two light treatments, between 16 and 24 weeks of age – that more active behaviors (locomotion, standing and foraging combined) were performed in the forest and daylight treatments. No differences were observed in resting or comfort behaviors (Wichman et al., 2021). It is, however, important to note that in their study the light treatments not only differed in UV but also in their color spectra, and that their behavioral observations covered a larger part of the day than the observations in the current study. Moreover, given that their implemented light intensity was much lower than the light intensity implemented in the current study (approximately 98 lux), the addition of UV light might have resulted in a relatively larger increase in visibility of the environment than in the current study. Rana et al. (2021) studied behavioral differences between light treatments (LED white light as control, visible spectrum plus infrared wavelengths, visible spectrum plus UVA wavelengths or visible spectrum plus UVA and UVB wavelengths; all at three levels of intensity) in adult laying hens. They observed, compared to the control light, less time spent feeding under UVA light, more foraging in the medium intensity of UVA with UVB light, more ground pecking in the low and medium intensity of UVA with UVB light, and more preening in the low intensity of UVA with UVB light. This contrasts with the lack of observed behavioral differences in the current study. However, in the study of Rana et al. (2021) birds were tested individually in two-hour sessions in a test setup and behavior was recorded continuously, which differs from the setup in the current study and may underlie the observed differences in findings. Possibly, in the current study the recorded behaviors appear to be performed similarly in both light treatments due to the observed preferences for high-UV versus low-UV not being very strong or complete (i.e., there appeared to nearly always be birds present on both sides of the pen according to the bird counts in Supplementary data 2). For future research, it is recommended to study behavior at the individual level (with individual identification of pullets) in group housing (to include potential social effects), to assess whether individuals select one of the two light conditions for performing specific behaviors.

#### Breed and age effects on behavior

It was observed that W birds were more active in the first week of age than at later ages or than B birds, and that B birds showed more standing behavior than W birds. This observation of W birds being more active than B birds aligns with other studies. Rentsch et al. (2023) studied the behavior of brown and white pullets and observed that white pullets generally exercised more frequently than brown pullets. The higher activity early in life may be linked to early exploration of the environment when the birds are young, after which they may become habituated to their surroundings and show less exploration behavior. In line with this, the proportion of foraging was higher in weeks 1 and 6 than in week 10 in W hens. More pecking at litter or enrichment may be conducted early in life to explore the surroundings. In B hens, a peak in foraging behavior was observed in week 4 (and a lower proportion of standing behavior for both breeds), and not in week 1 as for W birds. This again highlights that W and B may differ in their behavior over time, suggesting that housing and management may need to be adapted in a breed-specific manner. In addition, less ingestion behavior was observed early in life than at later ages. This contrasts with observations by Keeling et al. (2017), who studied layer pullets from 3 to 18 weeks of age and observed a decrease in feeding and drinking time with increasing age. However, they did not study the first 2 weeks of life, which was the time period in which the observed ingestion behavior was lowest in our study. We furthermore observed variation in comfort behavior (preening and dustbathing) over time, as well as differences between W and B birds. Possibly, this variation over time and breeds can be explained by the observation that preening and dustbathing are both behaviors that show strong behavioral synchrony in domestic fowl (Hoppitt et al., 2007). Perhaps if one bird starts preening or dustbathing, others will follow, and if this happens during a behavior observation (i.e., a scan sample), a relatively large number of animals will show comfort behavior at the same time, causing quite some variation between sampling days. This, however, requires further investigation.

## Conclusion

This study investigated the preference of layer pullets of two breeds for different levels of UVA light, and assessed under which light condition they spent most time and what behaviors were performed in the different light conditions. It was observed that W birds had a preference for high-UV light in the first three weeks of life when assessing the full day, while no clear preferences were observed for B birds. In the last hour before lights off and the first hour of the dark period, B showed a preference for low-UV in week 2 and no clear preference at later ages, while W birds did not show a clear preference at the end of the day at any age. No differences between the high-UV and low-UV light condition were observed in the proportion of behaviors performed in each. Overall, the observed preferences in this study varied between breeds and over time, and did not indicate an exclusive preference (i.e., there appeared to nearly always be birds present on both sides of the pen). This suggests that there might not be a one-size-fits-all approach to providing light conditions to laying hen pullets. Instead, offering a varied environment in time and space, by providing different light conditions at different times in the day and in different locations of the house, may offer the best opportunity for pullets to select their preferred environment, which may contribute to improving their welfare in production systems.

## CRediT authorship contribution statement

**Malou van der Sluis:** Writing – review & editing, Writing – original draft, Visualization, Formal analysis. **Jerine A.J. van der Eijk:** Writing – review & editing, Methodology, Conceptualization. **Thea G.C.M. van Niekerk:** Writing – review & editing, Methodology, Conceptualization. **Marjaneh Taghavi:** Writing – review & editing, Software, Investigation, Formal analysis, Data curation. **Dennis E. te Beest:** Writing – review & editing, Formal analysis. **Maaike Wolthuis-Fillerup:** Writing – review & editing, Investigation, Data curation. **Henk Gunnink:** Writing – review & editing, Investigation, Data curation. **Ingrid C. de Jong:** Writing – review & editing, Supervision, Project administration, Methodology, Funding acquisition, Conceptualization.

## Disclosures

The authors declare that they have no known competing financial interests or personal relationships that could have appeared to influence the work reported in this paper.

## Appendix Supplementary materials

Image, application 1

Image, application 2

## Footnotes

Supplementary material associated with this article can be found, in the online version, at doi:10.1016/j.psj.2026.106825.

## References

[bib0001] Bédécarrats, G. Y., .and C. Hanlon. 2017. Effect of lighting and photoperiod on chicken egg production and quality Pages 65-75 in Egg Innovations and strategies for improvements.

[bib0002] Bennett A.T.D., Cuthill I.C. (1994). Ultraviolet vision in birds: what is its function?. Vis. Res..

[bib0003] Bright A. (2007). Plumage colour and feather pecking in laying hens, a chicken perspective?. Br. Poult. Sci..

[bib0004] Brooks M.E., Kristensen K.., Benthem K.J.v., Magnusson A., Berg C.W., Nielsen A., Skaug H.J., Mächler M., Bolker B.M. (2017). glmmTMB balances speed and flexibility among packages for zero-inflated generalized Linear Mixed modeling. The R Journal.

[bib0005] Clark A.J., Harrison C.., Bragg A.J., House G.M., Stephan A.B., Arguelles-Ramos M., Ali A. (2024). Effect of interrupting the daily scotophase period on laying hen performance. Bone Health, Behavior, and Welfare; Part I: Bone Health. Poultry.

[bib0006] Hoppitt W., Blackburn L., Laland K.N. (2007). Response facilitation in the domestic fowl. Anim. Behav..

[bib0007] Janczak A.M., Riber A.B. (2015). Review of rearing-related factors affecting the welfare of laying hens. Poult. Sci..

[bib0008] Keeling L.J., Newberry R..C., Estevez I. (2017). Flock size during rearing affects pullet behavioural synchrony and spatial clustering. Appl. Anim. Behav. Sci..

[bib0009] Lenth R. V. 2.024. emmeans: estimated Marginal means, aka Least-Squares means. R package version 1.10.3 ed.

[bib0010] Liu K., Wang K., Fei T., Chai L., Xin H. (2018). ASABE Annual International Meeting.

[bib0011] Lyimo C.M., Weigend A.., Msoffe P.L., Eding H., Simianer H., Weigend S. (2014). Global diversity and genetic contributions of chicken populations from African, Asian and European regions. Anim. Genet..

[bib0012] Manet M.W.E., Kliphuis S., Nordquist R.E., Goerlich V.C., Tuyttens F.A.M., Rodenburg T.B. (2023). Brown and white layer pullet hybrids show different fear responses towards humans, but what role does light during incubation play in that?. Appl. Anim. Behav. Sci..

[bib0013] Muir W.I., Akter Y.., Kho S.K.Y., Bruerton K., Groves P.J. (2024). The impact of lighting regimen and feeding program during rearing on hy-line brown pullets at the end of rearing and during early lay. Animals.

[bib0014] Poudel I., Beck M.M., Kiess A.S., Adhikari P. (2022). The effect of blue and red LED light on the growth, egg production, egg quality, behavior, and hormone concentration of Hy-Line W-36 laying hens. J. Appl. Poult. Res..

[bib0015] Prescott N.B., Wathes C.M. (1999). Reflective properties of domestic fowl (Gallus g. domesticus), the fabric of their housing and the characteristics of the light environment in environmentally controlled poultry houses. Br. Poult. Sci..

[bib0016] Prescott N.B., Wathes C.M. (1999). Spectral sensitivity of the domestic fowl (Gallus g. domesticus). Br. Poult. Sci..

[bib0017] R Core Team (2024). R: A Language and Environment for Statistical Computing.

[bib0018] Rana M.S., Campbell D.L.M. (2021). Application of ultraviolet light for poultry production: a review of impacts on behavior. Physiol., Produc. Front. Anim. Sci..

[bib0019] Rana M.S., Cohen-Barnhouse A..M., Lee C., Campbell D.L.M. (2021). Preference testing for UV light spectrum and intensity in laying hens. Poult. Sci..

[bib0020] Rana M.S., Lee C.., Walkden-Brown S.W., Campbell D.L.M. (2024). Effects of ultraviolet light supplementation on hen behaviour and welfare during early lay. Appl. Anim. Behav. Sci..

[bib0021] Rentsch A.K., Harlander A.., Niel L., Siegford J.M., Widowski T.M. (2024). Raising laying hens: housing complexity and genetic strain affect startle reflex amplitude and behavioural response to fear-inducing stimuli. R. Soc. Open. Sci..

[bib0022] Rentsch A.K., Harlander A.., Siegford J.M., Vitienes I., Willie B.M., Widowski T.M. (2023). Rearing laying hens: the effect of aviary design and genetic strain on pullet exercise and perching behavior. Front. Anim. Sci..

[bib0023] Sadvakassova G., Ghaly M., Chew J.A., Poorhemati H., Beaulac K., Shynkaruk T., Widowski T., Schwean-Lardner K., Komarova S.V. (2022). Research note: effect of light intensity of calcium homeostasis in pullets. Poult. Sci..

[bib0024] Sobotik E.B., Nelson J..R., Archer G.S. (2020). How does ultraviolet light affect layer production, fear, and stress. Appl. Anim. Behav. Sci..

[bib0025] Stadig L.M., Rodenburg T..B., Ampe B., Reubens B., Tuyttens F.A.M. (2017). Effect of free-range access, shelter type and weather conditions on free-range use and welfare of slow-growing broiler chickens. Appl. Anim. Behav. Sci..

[bib0026] van der Eijk J.A.J., Izquierdo Garcia-Faria T., Melis S., van Riel J.W., Te Beest D.E., de Jong I.C. (2025). Light intensity preferences of broiler chickens is affected by breed, age, time of day and behaviour. Sci. Rep..

[bib0027] van der Sluis M., van der Eijk J.A.J., Bekhit R., te Beest D.E., Gunnink H., Melis S., de Jong I.C. (2025). Ultraviolet light provisioning: preferences from the broilers’ viewpoint. Appl. Anim. Behav. Sci..

[bib0028] van der Sluis M., van der Eijk J.A.J., Izquierdo Garcia-Faria T., te Beest D.E., Wolthuis-Fillerup M., de Jong I.C. (2025). Light spectrum and intensity preferences of fast- and slower-growing broilers vary by age, behaviour and time of day. Appl. Anim. Behav. Sci..

[bib0029] Wei Y., Zheng W., Tong Q., Li Z., Li B., Shi H., Wang Y. (2022). Effects of blue-green LED lights with two perceived illuminance (human and poultry) on immune performance and skeletal development of layer chickens. Poult. Sci..

[bib0030] Wichman A., De Groot R., Hastad O., Wall H., Rubene D. (2021). Influence of different light spectrums on behaviour and welfare in laying hens. Animals.

[bib0031] World Health Organization (2016). Radiation: ultraviolet (UV) radiation. https://www.who.int/news-room/questions-and-answers/item/radiation-ultraviolet-(uv)#:~:text=UV/20levels/20are/20higher/20closer,UV/20radiation/20can/20be/20absorbed.&text=With/20increasing/20altitude/20less/20atmosphere/20is/20available/20to/20absorb/20UV/20radiation.

