# Supplementary material for: Ultraviolet light preferences in a white and a brown layer pullet strain
Source: Poult Sci. 2026 Mar 20;105(6):106825. doi: 10.1016/j.psj.2026.106825 (PMC13067110; doi:10.1016/j.psj.2026.106825)
Supplement: Supplementary file 1 [file mmc1.docx]

**Supplementary data 1: Measured lux intensities and spectra for the low-UV and high-UV light conditions**

The measured lux intensities per light area (i.e. low-UV or high-UV) are given in **Table S1**.

**Table S1. Measured light intensities (in lux) per light area (i.e. low-UV or high-UV).**

| Location | Low-UV | High-UV | Average (range) |  |
| --- | --- | --- | --- | --- |
| Centre | 157.3 (149.4-164.9) | 159.9 (158.0-165.4) | 158.6 (149.4-165.4) |  |
| Corner | 36.4 (29.7-48.4) | 36.8 (29.4-44.3) | 36.6 (29.4-48.4) |  |
| Average | 96.9 | 98.4 | 97.6 |  |

**Figure S1** shows the light spectra of the low-UV and the high-UV light condition.


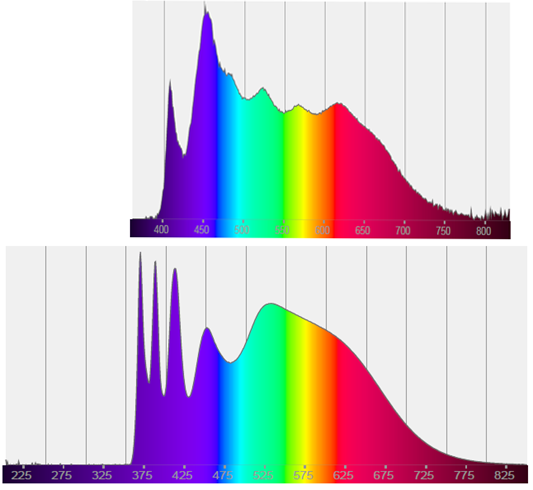


**Figure 1**. Spectral wavelengths for top) low-UV and bottom) high-UV light. The x-axis ranges differ between the two spectra.
