# Supplementary material for: Ultraviolet light preferences in a white and a brown layer pullet strain
Source: Poult Sci. 2026 Mar 20;105(6):106825. doi: 10.1016/j.psj.2026.106825 (PMC13067110; doi:10.1016/j.psj.2026.106825)
Supplement: Supplementary file 2 [file mmc2.docx]

**Supplementary data 2: Variation in light preference**

**Figures S2.1** and **S2.2** show for Lohmann Classic Brown and Lohmann LSL Classic birds, respectively, the relative proportions of birds present in the different light treatment compartments over time.


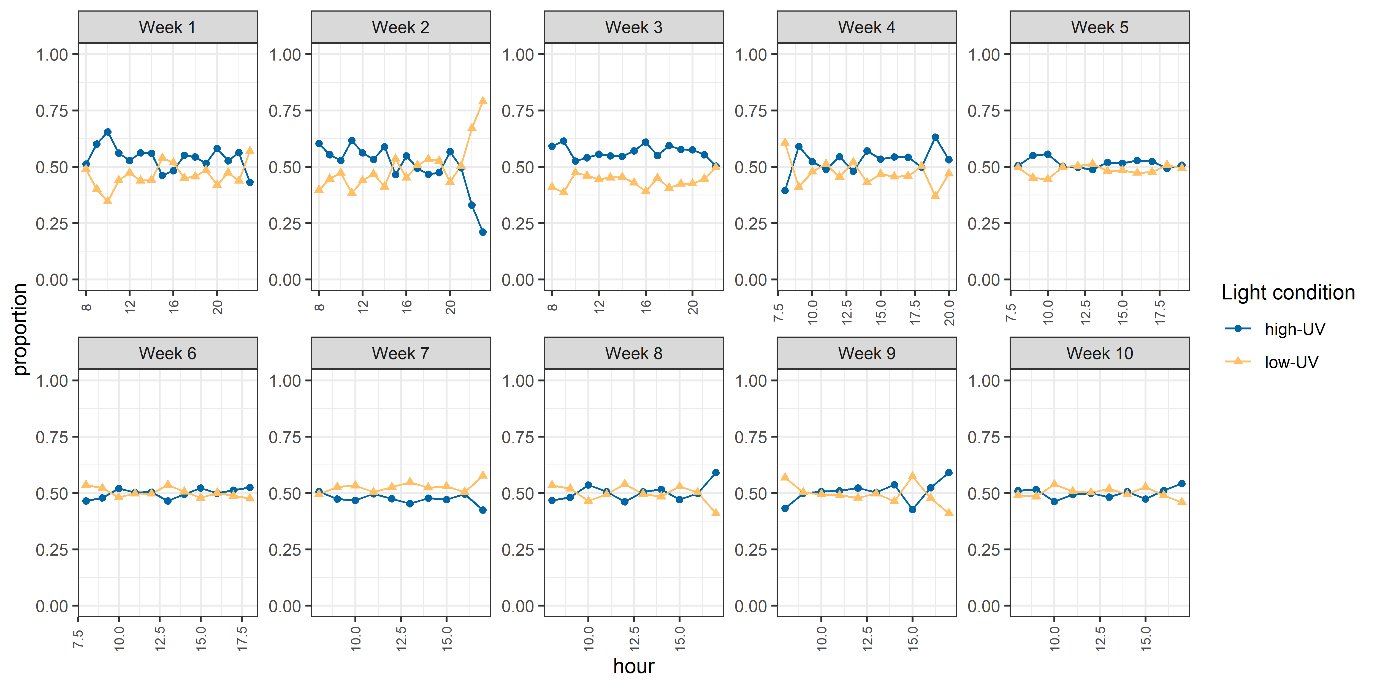


**Figure S2.1: Relative proportions of Lohmann Classic Brown birds present in the different light treatment compartments over time.** Different panels show the different weeks of age, and the hour of the day is shown on the x-axis. Note that the start hour is indicated on the x-axis, e.g. hour 8 is the period from 08:00 to 09:00. High-UV = high level of UV light present; low-UV = low level of UV light present.

**
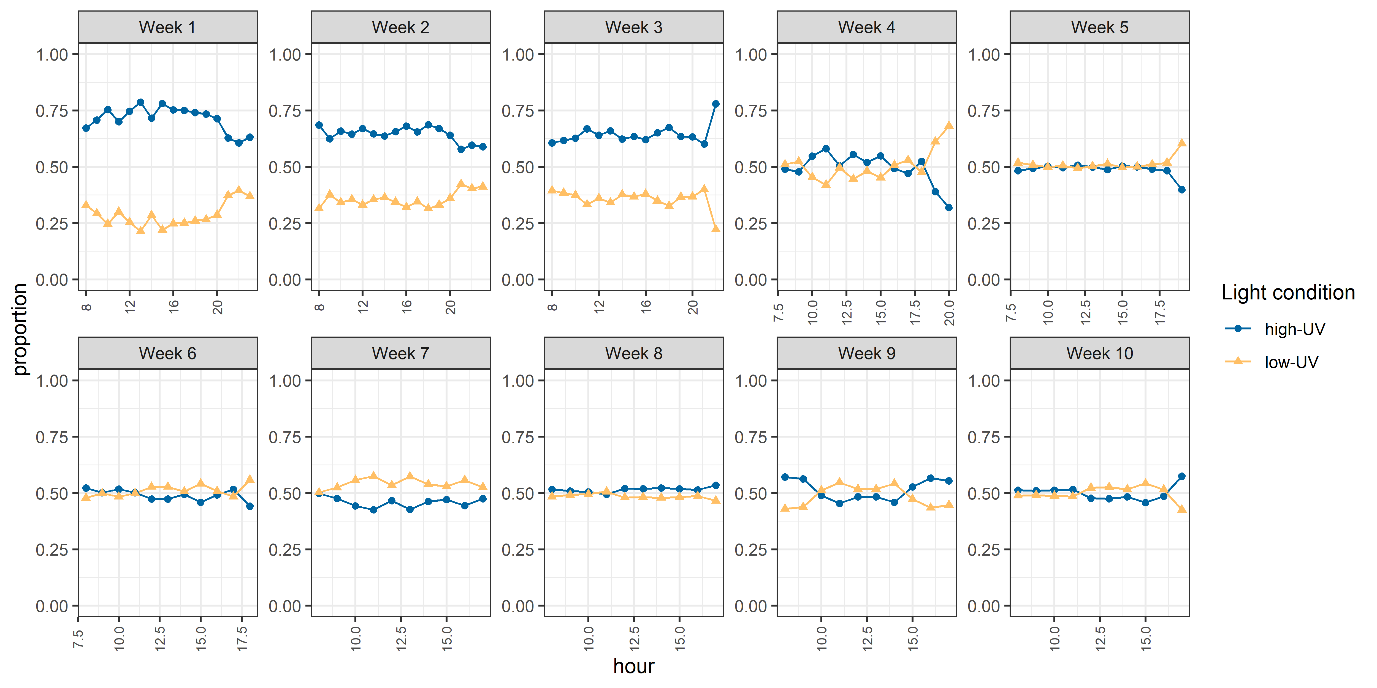
**

**Figure S2.2: Relative proportions of Lohmann LSL Classic birds present in the different light treatment compartments over time.** Different panels show the different weeks of age, and the hour of the day is shown on the x-axis. Note that the start hour is indicated on the x-axis, e.g. hour 8 is the period from 08:00 to 09:00. High-UV = high level of UV light present; low-UV = low level of UV light present.

**Figures S2.3** and **S2.4** show for Lohmann Classic Brown and Lohmann LSL Classic birds, respectively, the variation between pens in location counts over weeks.


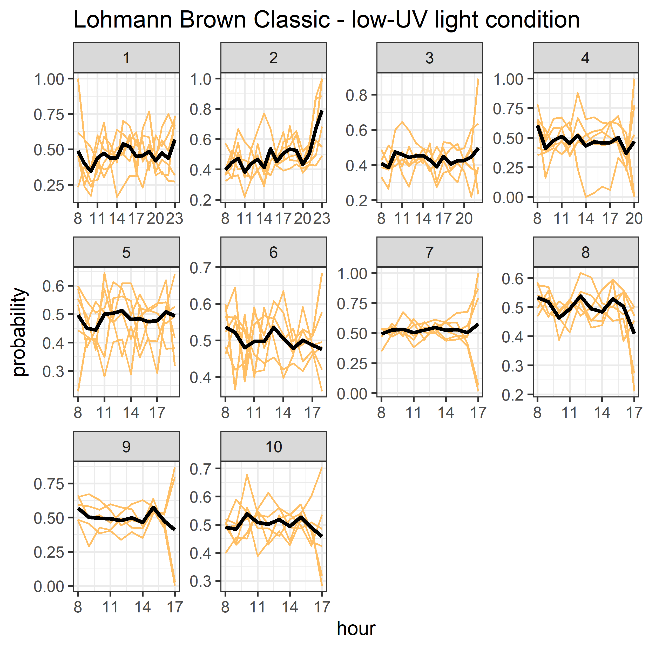

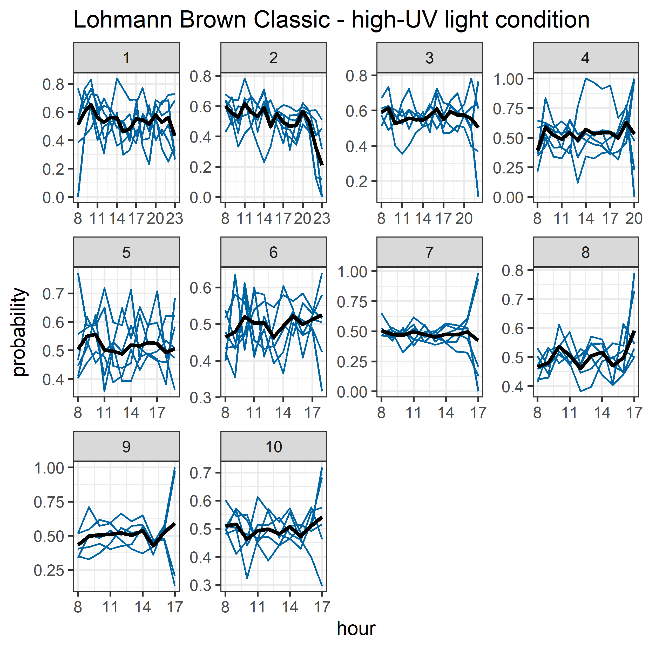


**Figure S2.3: Proportional bird counts in the two light conditions for Lohmann Classic Brown birds over time.** Separate panels show different weeks, with lines representing individual pens. Black lines indicate the mean across pens.


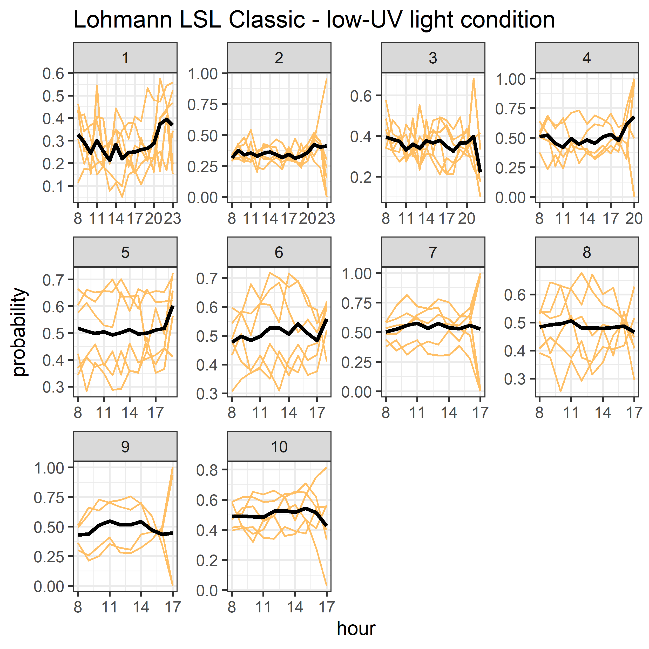

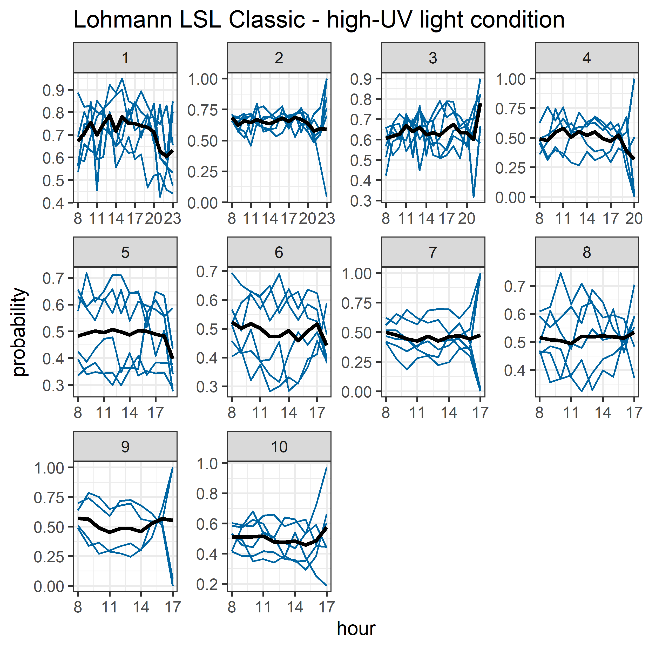


**Figure S2.4: Proportional bird counts in the two light conditions for Lohmann LSL Classic birds over time.** Separate panels show different weeks, with lines representing individual pens. Black lines indicate the mean across pens.
